# DWARF14, A Receptor Covalently Linked with the Active Form of Strigolactones, Undergoes Strigolactone-Dependent Degradation in Rice

**DOI:** 10.3389/fpls.2017.01935

**Published:** 2017-11-09

**Authors:** Qingliang Hu, Yajun He, Lei Wang, Simiao Liu, Xiangbing Meng, Guifu Liu, Yanhui Jing, Mingjiang Chen, Xiaoguang Song, Liang Jiang, Hong Yu, Bing Wang, Jiayang Li

**Affiliations:** ^1^State Key Laboratory of Plant Genomics and National Center for Plant Gene Research (Beijing), Institute of Genetics and Developmental Biology, Chinese Academy of Sciences, Beijing, China; ^2^University of Chinese Academy of Sciences, Beijing, China

**Keywords:** *Oryza sativa*, plant hormones, strigolactones, hydrolase, DWARF14, proteolysis, signal transduction

## Abstract

Strigolactones (SLs) are the latest confirmed phytohormones that regulate shoot branching by inhibiting bud outgrowth in higher plants. Perception of SLs depends on a novel mechanism employing an enzyme-receptor DWARF14 (D14) that hydrolyzes SLs and becomes covalently modified. This stimulates the interaction between D14 and D3, leading to the ubiquitination and degradation of the transcriptional repressor protein D53. However, the regulation of SL perception in rice remains elusive. In this study, we provide evidences that D14 is ubiquitinated after SL treatment and degraded through the 26S proteasome system. The Lys280 site of the D14 amino acid sequence was important for SL-induced D14 degradation, but did not change the subcellular localization of D14 nor disturbed the interaction between D14 and D3, nor D53 degradation. Biochemical and genetic analysis indicated that the key amino acids in the catalytic center of D14 were essential for D14 degradation. We further showed that D14 degradation is dependent on D3 and is tightly correlated with protein levels of D53. These findings revealed that D14 degradation takes place following D53 degradation and functions as an important feedback regulation mechanism of SL perception in rice.

## Introduction

Strigolactones (SLs), a group of carotenoid-derived terpenoid lactones produced by plants, were initially characterized as signals that enable parasitic plants to detect their host (Cook et al., 1966), and also as signals recognized by arbuscular mycorrhizal (AM) fungi in the rhizosphere to build the symbiotic association with host plants (Akiyama et al., 2005). Besides the roles of SLs in the rhizosphere, SLs have been identified as endogenous phytohormones that are transported from roots to shoots and suppress shoot branching by inhibiting the outgrowth of axillary buds (Beveridge et al., 1996; Foo et al., 2001; Booker et al., 2005; Gomez-Roldan et al., 2008; Umehara et al., 2008). In addition, SLs have profound effects in many aspects of plant development, including internode elongation, leaf shape and senescence, shoot gravitropism, stem secondary thickening, root architecture, and the drought tolerance (Al-Babili and Bouwmeester, 2015; Waters et al., 2017).

The key components required for SL biosynthesis and signaling have been identified from genetic characterizations of highly branched mutants, including *ramosus* (*rms*) in pea (*Pisum sativum*), *more axillary growth* (*max*) in *Arabidopsis thaliana*, *decreased apical dominance* (*dad*) in petunia (*Petunia hybrida*), and *dwarf* (*d*) or *high-tillering dwarf* (*htd*) in rice (*Oryza sativa*). In the SL biosynthetic pathway, SLs are derived from all-*trans*-β-carotene, which is converted to 9-*cis*-β-carotene by the isomerase DWARF27 (D27) (Lin et al., 2009; Alder et al., 2012), and subsequently catalyzed into carlactone by carotenoid cleavage oxygenase 7 (CCD7) and CCD8 (Beveridge et al., 1996; Sorefan et al., 2003; Booker et al., 2004; Seto et al., 2014). The subsequent catalytic reactions are diverse in different species. In Arabidopsis, the cytochrome P450 enzyme MAX1 converts carlactone to carlactonoic acid (CLA), which is further converted to a methyl carlactonoate (MeCLA) by an unknown enzyme (Abe et al., 2014). Subsequently, LATERAL BRANCHING OXIDOREDUCTASE (LBO) converts MeCLA to an unknown SL-like product (MeCLA+16 Da) (Brewer et al., 2016). In rice, the *MAX1* homolog *Os01g0700900* is responsible for the oxidation of carlactone to 4-deoxyorobanchol (4DO), while another *MAX1* homolog, *Os01g0701400*, functions as an orobanchol synthase that converts 4DO to orobanchol (Zhang et al., 2014). In sorghum, a newly identified sulfotransferase LOW GERMINATION STIMULANT1 (LGS1) is responsible for a change of the dominant SL in root exudates from 5-deoxystrigol to orobanchol via an unknown mechanism and regulates the *Striga* resistance (Gobena et al., 2017).

In SL signaling, three key components have been identified from genetic screen of SL-insensitive mutants in rice, Arabidopsis, pea, and petunia, which include the α/β-fold hydrolase D14/AtD14/RMS3/DAD2, the F-box protein D3/MAX2/RMS4/PhMAX2A, and the repressor proteins D53/D53-Like SMXLs (Stirnberg et al., 2002, 2007; Ishikawa et al., 2005; Arite et al., 2009; Gao et al., 2009; Liu et al., 2009; Hamiaux et al., 2012; Waters et al., 2012; Jiang et al., 2013; Nakamura et al., 2013; Zhou et al., 2013; Soundappan et al., 2015; Wang et al., 2015). Perception of SLs depends on a novel mechanism involving the formation of a covalently linked intermediate molecule (CLIM) from the binding of a SL molecule to the receptor D14 and being hydrolyzed by D14. This reaction promotes a conformational change of D14, leading to the interaction between D14 and D3 and triggering the SL signal transduction (Yao et al., 2016). S Ls can also induce the interaction between D14 and D53, leading to ubiquitination and degradation of D53 in a D3- and D14-dependent manner (Jiang et al., 2013; Zhou et al., 2013). D53 contains three ethylene-responsive element binding factor-associated amphiphilic repression (EAR) motifs, which are essential to recruit the transcriptional co-repressor TOPLESS (TPL) and TPL-related proteins (TPRs) and could potentially repress the activities of target transcription factors (Jiang et al., 2013). Recently, Ideal Plant Architecture1 (IPA1), a key regulator of plant architecture in rice, has been identified as one of the long-speculated transcription factors involved in SL signaling. IPA1 can physically interact with D53 and plays an essential role in the feedback regulation of SL-induced *D53* expression (Song et al., 2017).

Although the “de-repression activation” mechanism in SL signaling is similar to the signaling pathways of auxin, gibberellic acid (GA) and jasmonic acid (JA) (Dharmasiri et al., 2005; Kepinski and Leyser, 2005; Ueguchi-Tanaka et al., 2005; Chini et al., 2007; Thines et al., 2007; Jiang et al., 2013; Zhou et al., 2013), the SL receptor D14 functions as a non-canonical receptor (Yao et al., 2016). Therefore, the molecular mechanism underlying the inactivation of SL signaling triggered by the D14-SCF^D3^-D53 complex becomes an important open question. The degradation of signal receptors by the 26S proteasome system is critical for fine-tuning on signal transduction of plant hormones and environmental signals, such as JA, abscisic acid (ABA), ethylene and the blue light (Chen et al., 2007; Kevany et al., 2007; Yan et al., 2013; Kong et al., 2015; Tao et al., 2015; Liu et al., 2016). In *Arabidopsis thaliana*, AtD14 has been shown to undergo 26S proteasome-dependent degradation, but the molecular mechanism of AtD14 degradation remains elusive (Chevalier et al., 2014). In rice, whether D14 undergoes degradation and the regulatory mechanism underlies SL perception remain unknown. Due to the profound effects of SLs on tillering, a key agronomic trait in cereal crops, investigating the regulation of SL perception is important for improving plant architecture and grain yield of rice. In this study, we show that SLs stimulate the ubiquitination and degradation of D14 through 26S proteasome in rice. A point mutation at Lys280 of D14 could greatly impair D14 degradation, but has little effect on SL signal transduction. We also show that the hydrolase activity of D14 and the intact functions of D3 and D53 are both required for SL-induced D14 degradation. These discoveries have paved a way for elucidating of the inactivation mechanism of the SL perception in rice.

## Materials and Methods

### Plant Materials

Rice (*Oryza sativa* L. spp. *japanica*) mutants used in this study were *d53* and *d14* from Nipponbare and *d3* from Zhonghua 11 (ZH11) as described previously (Jiang et al., 2013; Sang et al., 2014). Rice plants were cultivated in the experimental field of the Institute of Genetics and Developmental Biology at Beijing in the summer and Hainan in the winter. For quantitative PCR with reverse transcription and transient expression analysis, the seedlings of wild-type and mutants were grown in a growth chamber with a 16-h light at 28°C and 8-h dark at 25°C photoperiod with approximately 200 μM m^-2^ s^-1^ photon density and 70% humidity. For calli treatment assays, rice calli were cultured on selection medium (Gamborg et al., 1968; Chu et al., 1975) for 7 days at 28°C in a light-avoided environment in greenhouse.

### Chemicals and Reagents

The synthetic SL analog GR24, a racemic mixture (*rac*-GR24) comprising amounts of GR24^5DS^ and GR24^ent-5DS^, was the product from Chiralix, MG132 from Calbiochem, the complete protease inhibitor cocktail (Cat#04693132001) and anti-GFP antibody (Cat#11814460001) from Roche, anti-actin antibody (Cat#M20009L) from Abmart, anti-Flag antibody (Cat#M20008) from Abmart, GFP-Trap^®^A beads (Cat#120716001A) from Chromotek, TRIzol kit (Cat#15596018) and Superscript III RT kit (Cat#18080-051) from Invitrogen, TURBO DNA-free^TM^ Kit (Cat#AM1907) from Ambion, SsoFast EvaGreen supermix (Cat#172-5201AP) from Bio-Rad, and Glutathione Sepharose 4 Fast Flow (Cat#17-5132-01) from GE healthcare. The anti-ubiquitin and anti-D53 antibodies are generated as described previously (Jiang et al., 2013; Tian et al., 2015).

### Plasmid Construction

The plasmids of *Actin*:*D14-GFP*, *Actin:D14^S147A^-GFP* and *Actin:D14 ^H297Y^-GFP* were generated in previous studies (Jiang et al., 2013). To construct the *35S:D14-GF*P plasmid, the coding sequence of *D14* was amplified with primers of pBI221*-*D14*-*F/R (Supplementary Table 1) and recombined into pBI221-GFP vector. To generate the *35S:D14^K280E^-GFP* plasmid, the full-length of *D14^K280E^* coding sequences were derived from *35S:D14-GFP* plasmid by site-directed mutagenesis using the primers of 35S-D14^K280E^-F/R (Supplementary Table 1), and subsequently cloned into the pBI221-GFP vector. To construct the plasmid of *D14-GFP*, the coding sequence of *D14* was amplified with primers of Ubi-D14-F/R (Supplementary Table 1) and cloned into pTCK303. To generate the plasmids of *D14^K33E^-GFP*, *D14^K55E^-GFP*, *D14^K166E^-GFP*, *D14^K246E^-GFP* and *D14^K280E^-GFP*, the full-length of the coding sequences of *D14^K33E^*, *D14^K55E^*, *D14^K166E^*, *D14^K246E^* and *D14^K280E^* were derived from *35S:D14-GFP* by site-directed mutagenesis using primers of D14^K33E^-F/R, D14^K55E^-F/R, D14^K166E^-F/R, D14^K246E^-F/R and D14^K280E^-F/R (Supplementary Table 1), respectively, and subsequently recombined into the binary vector pTCK303.

### Chemical Treatment of Rice Calli

To examine whether SLs could induce the degradation of D14-GFP, D14^K33E^-GFP, D14^K55E^-GFP, D14^K166E^-GFP, D14^K246E^-GFP, and D14^K280E^-GFP, calli of these transgenic lines were cultured on the selection medium (Gamborg et al., 1968; Chu et al., 1975) for 7 days at 28°C and then transferred into a liquid medium. After the treatment with *rac-*GR24 at the indicated concentration for various hours, calli were collected and frozen at -80°C. Total proteins were extracted with the extraction buffer (50 mM sodium phosphate buffer, pH 7.0, 150 mM NaCl, 10% (v/v) glycerol, 0.1% NP-40, and 1× complete protease inhibitor cocktail). The supernatant was boiled with 5× SDS buffer at 100°C for 10 min and immunoblotting was performed with anti-GFP and anti-Actin antibodies.

### *In Vivo* Ubiquitination Assay

The seedlings of wild-type were cultured in greenhouse for 2 weeks. Protoplasts were prepared from shoot tissues and transformed with *35S:D14-GFP* as described (Bart et al., 2006). After incubation at 28°C for 12 h in W5 solution (154 mM NaCl, 125 mM CaCl_2_, 5 mM KCl, 2 mM MES pH 5.7), the protoplasts were collected and pretreated with 50 μM MG132 for 1 h and then treated with 20 μM *rac-*GR24 or DMSO at 28°C for 3 h. Total proteins were extracted with the extraction buffer (50 mM sodium phosphate buffer, pH 7.0, 150 mM NaCl, 10% (v/v) glycerol, 0.1% NP-40, 50 mM MG132, and 1× complete protease inhibitor cocktail). The lysates were centrifuged at 18,000 *g* for 20 min at 4°C. The supernatant was then taken for immunoprecipitation and subsequent immunoblot analysis using anti-ubiquitin and anti-GFP antibodies. Under the guidance of the supplier’s instruction, 20 μL of GFP-Trap^®^A beads were added into 1.2 mL totally extracted proteins and incubated at 4°C for 3 h with gentle rotation. The beads were washed three times with the washing buffer without NP-40 and then boiled with 50 μL SDS-PAGE sample buffer for protein blotting. Mouse anti-ubiquitin monoclonal antibody was used at a 1:2,000 dilution and mouse anti-GFP polyclonal antibodies at a 1:3,000 dilution.

### Gene Expression Analysis

The seedlings of wild-type were hydroponic-cultured (pH 5.5) in greenhouse for 2 weeks. After 5 μM *rac*-GR24 treatment, the shoot base (0.5 cm) of the seedlings were harvested at different time points, total RNAs were extracted using a TRIzol kit according to the manufacturer’s manual and then treated with the TURBO DNA-free^TM^ Kit and used for complementary DNA synthesis with the Superscript III RT kit. About 12.5 μg total RNA was added into a 20-μL TURBO DNase mixture reaction system and incubated at 37°C for 30 min, and then added 2 μL DNase inactivation reagent and centrifuged at 12,000 *g* for 10 min, and finally 4 μL of the supernatants was used for complementary DNA synthesis. The quantitative PCR with reverse transcription experiments were performed with gene-specific primers of D14-RT-F/R and D53-RT-F/R (Supplementary Table 1) on a CFX 96 real-time PCR detection system (Bio-Rad). Each reaction volume was set as 10 μL, consisting of 5 μL SsoFast EvaGreen supermix, 0.5 μL sense primer (5 μM), 0.5 μL antisense primer (5 μM), 2.0 μL diluted cDNA, and 2 μL ddH_2_O. Rice *UBIQUITIN* (LOC_Os03g13170) gene was used as the internal control.

### Co-IP Assay

The seedlings of the wild type were hydroponic-cultured (pH 5.5) in greenhouse for 2 weeks. Protoplasts generated from shoot tissues were transformed with *35S:D14-Flag*, *35S:D3-GFP*, or *35S-GFP* (Bart et al., 2006). After incubation at 28°C for 12 h in W5 solution (154 mM NaCl, 125 mM CaCl_2_, 5 mM KCl, 2 mM MES, pH 5.7), proteins were extracted from the collected protoplasts using the extraction buffer (50 mM sodium phosphate buffer, pH 7.0, 150 mM NaCl, 10% (v/v) glycerol, 0.1% NP-40, 50 μM MG132, 1× complete protease inhibitor cocktail) by centrifugation at 20,000 *g* for 20 min at 4°C and then the supernatant was taken out for Co-IP experiments. Then 20 μL of GFP-Trap^®^A beads was added into 1.2 mL extracted total proteins and incubated at 4°C for 3 h with gentle rotation in the presence or absence of 10 μM *rac*-GR24. The beads were washed three times with the washing buffer lack of NP-40 and then boiled with 50 μL SDS-PAGE sample buffer for protein blot. The proteins of D3-GFP and GFP were detected by mouse anti-GFP antibody at a 1:3,000 dilution, and the D14-Flag proteins by mouse anti-FLAG antibody at a 1:2,000 dilution.

### Microscopy Analyses

Protoplasts were prepared from shoot tissues of 2-week-old seedlings and transformed with the plasmid of *35S:GFP*, *35S:D14-GFP* or *35S:D14^K280E^-GFP*. The SV40NLS-mCherry plasmid, which bears a strong nucleus localization signal peptide, was co-transformed into the protoplasts to label the nucleus (Ye et al., 2012). After incubation for 14 h at 28°C in the dark, the protoplasts were collected to observe the GFP and mCherry signals with confocal microscope at the excitation wavelengths of 488 and 559 nm, respectively (FluoView FV1000; Olympus).

## Results

### SLs Stimulate the Ubiquitination and Degradation of D14

**FIGURE 1:**
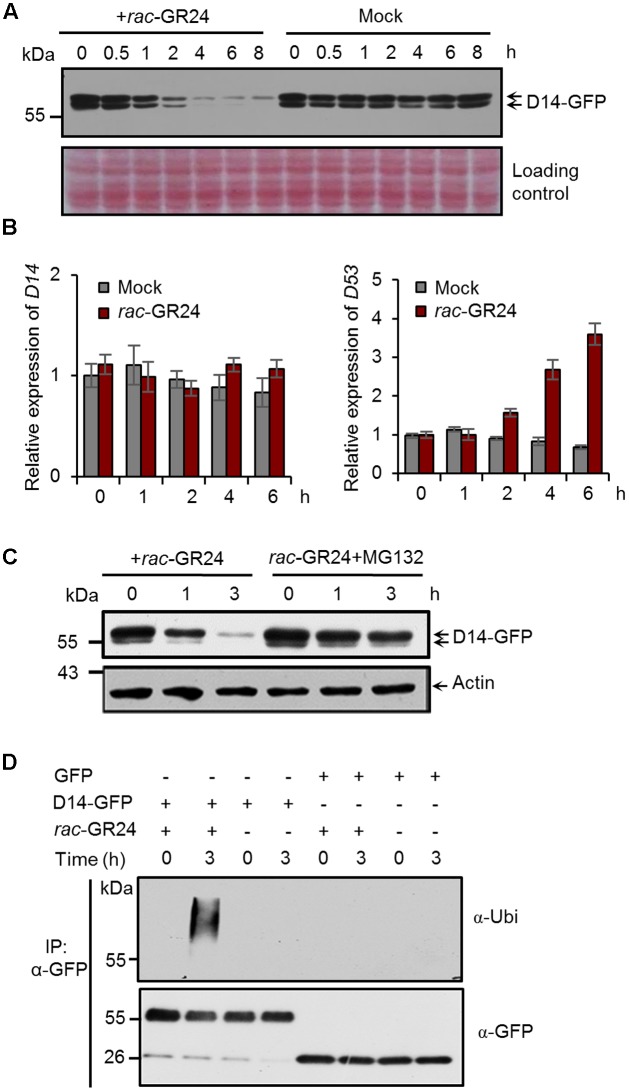
Strigolactones (SLs) promote ubiquitination and degradation of D14 in rice. **(A)** Protein levels of D14-GFP in calli of the *Act:D14-GFP/d14* transgenic line at different time points after 20 μM *rac*-GR24 treatment with DMSO as a control. D14-GFP was detected by immunoblotting with an anti-GFP monoclonal antibody. Relative protein levels were determined by densitometry and normalized to loadings determined by Ponceau staining (red) in the immunoblotting analyses. **(B)** Relative expression levels of *D14* and *D53* in 2-week-old seedlings after 5 μM *rac*-GR24 treatment. Values represent means ± SEM, *n* = 3. **(C)** D14-GFP protein levels in calli of the *D14-GFP/d14* transgenic line at different time points after *rac*-GR24 treatment in the presence or absence of MG132. Calli are pretreated with 50 μM MG132 for 1 h and then treated with 20 μM *rac*-GR24 or DMSO for 3 h. D14-GFP was detected by immunoblotting with an anti-GFP monoclonal antibody. Actin1 were used as loading control in the immunoblotting analyses. **(D)** Ubiquitination analysis of D14-GFP in rice protoplasts. Rice (Nipponbare) protoplasts were transformed with *35S:D14-GFP* plasmids and incubated for 12 h, then pretreated with 50 μM MG132 for 1 h and immediately treated with 20 μM *rac*-GR24 or DMSO for 3 h. Proteins were extracted for affinity purification with an agarose-immobolized anti-GFP monoclonal antibody and followed by immunoblotting analysis with an anti-ubiquitin (upper panel) or anti-GFP (lower panel) monoclonal antibody.

To explore whether the SL receptor undergoes a feedback regulation in rice, we first investigated the D14 protein levels after SL treatment. In *Actin:D14-GFP* transgenic calli treated with 20 μM *rac*-GR24, the D14-GFP fusion protein amount begun to decrease at 1 h, and dramatically reduced at 4 h (**Figure 1A**). We then examined the expression levels of *D14* and *D53* upon 5 μM *rac*-GR24 treatment in 2-week-old seedlings, and found that *D14* transcripts were unaffected within 6 h treatment, while *D53* transcripts were strongly induced after 2 h treatment (**Figure 1B**). These results indicate that SLs could induce the degradation of the D14 protein but have no effect on *D14* transcription.

To investigate whether D14 is degraded through the ubiquitin-26S proteasome system, we detected D14 protein levels in the *Actin:D14-GFP* transgenic calli treated with 20 μM *rac*-GR24 in the presence or absence of MG132, and found that the SL-induced D14 degradation was strongly inhibited by MG132, indicating that the 26S proteasome pathway is involved in the degradation of D14 (**Figure 1C**). We further examined the polyubiquitination of D14 upon *rac*-GR24 treatment in rice protoplasts transformed with *35S:D14-GFP*. After 3 h treatment, the transiently expressed D14-GFP recombinant protein was polyubiquitinated, but no polyubiquitination signal was detected in any other negative control (**Figure 1D**). Collectively, these data demonstrate that SLs stimulate the ubiquitination and degradation of D14 via the ubiquitin-26S proteasome system in rice.

### Identification of Key Amino Acids Responsible for SL-Induced D14 Degradation

**FIGURE 2:**
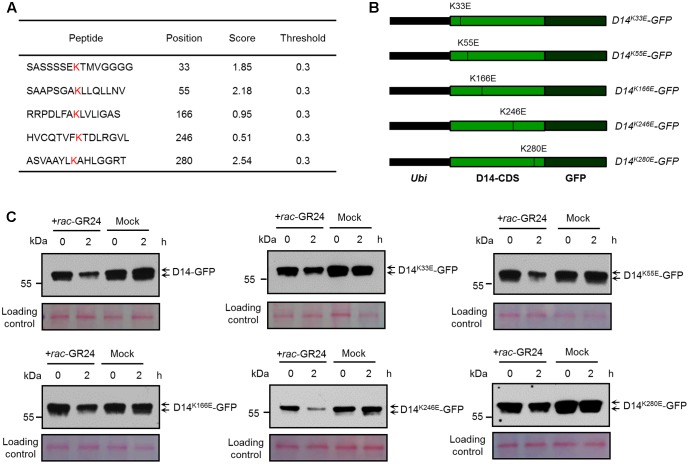
Characterization of key amino acid residues in D14 for its degradation. **(A)** The lysine sites in the D14 amino acid sequence and their predicted scores from the BDM-PUB website. **(B)** Schematic diagrams showing the constructs of D14 with point mutation of candidate ubiquitination sites. **(C)** Protein levels of D14-GFP, D14^K33E^-GFP, D14^K55E^-GFP, D14^K166E^-GFP, D14^K246E^-GFP and D14^K280E^-GFP in rice protoplasts transformed with plasmids shown in **(B)** after treatment with 10 μM *rac*-GR24 or DMSO for 2 h. Protein levels were detected by immunoblotting with an anti-GFP monoclonal antibody. Relative protein levels were normalized to the loading control determined by Ponceau staining (red).

To identify the potential ubiquitination sites of D14, we analyzed the protein sequence of D14 using BDM-PUB^1^. Five lysine sites, K33, K55, K166, K246, and K280, were predicted as potential candidate ubiquitination sites of D14, of which K280 has the highest score (**Figure 2A**). We then tested whether the degradation of D14 is affected when each ubiquitination site was mutated through creating the point-mutation constructs containing *D14^K33E^-GFP*, *D14^K55E^-GFP*, *D14^K166E^-GFP*, *D14^K246E^-GFP* or *D14^K280E^-GFP* and expressed them in rice protoplasts, respectively (**Figure 2B**). After treatment with 10 μM *rac*-GR24 for 2 h, these recombinant proteins showed degradation at different extents. Compared with the wild-type D14-GFP, the degradation of D14^K280E^-GFP was severely impaired and the degradation of D14^K33E^-GFP was moderately decreased upon SL treatment, but the degradation of D14^K55E^-GFP, D14^K166E^-GFP or D14^K246E^-GFP was relatively unaffected (**Figure 2C**), suggesting that K280 might be the key amino acid for SL-induced D14 degradation.

AtD14 has been reported to be degraded after SL treatment in *Arabidopsis thaliana* (Chevalier et al., 2014). We then compared the amino acid sequences of D14 with its orthologues including AtD14 from Arabidopsis, RMS3 from pea and DAD2 from petunia, and found that the K280 site of D14 is conserved in pea and petunia, but changes to Arginine in Arabidopsis (Supplementary Figure 1), suggesting that the mechanism of D14 degradation might be different between rice and Arabidopsis. Further analysis on the destabilizing effects of the K280E mutation indicate that no obvious conformational change occurs in D14^K280E^ based on structural prediction and comparison (Supplementary Figure 2). Taken together, these results indicate that the ubiquitination and degradation of D14 may involve multiple amino acids, of which K280 appears to play a major role.

### D14^K280E^ Mutation Does Not Affect SL-Induced D53 Degradation

**FIGURE 3:**
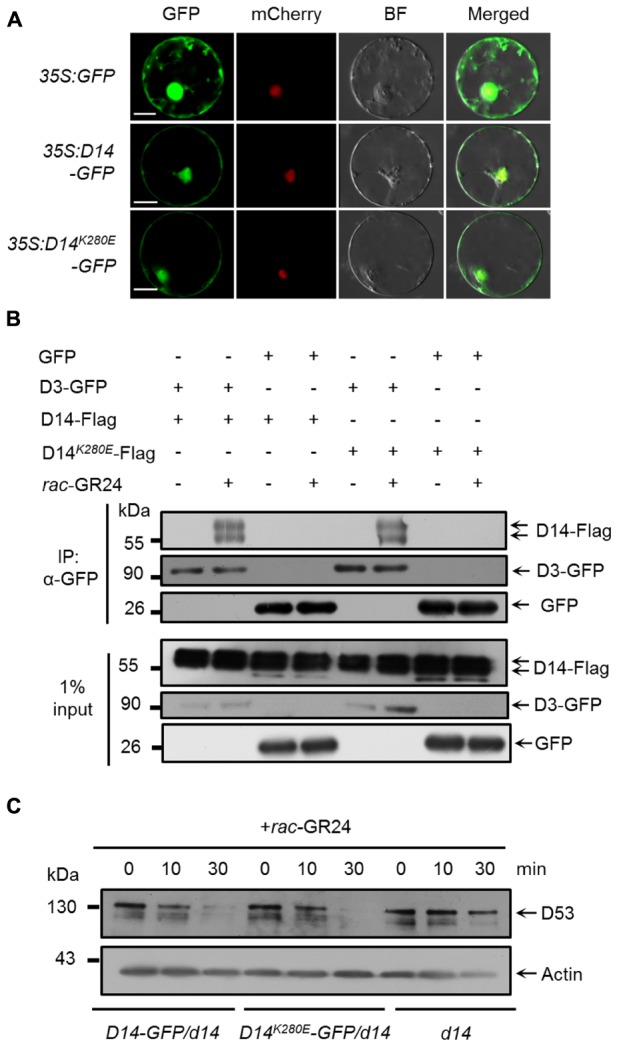
Effect of D14^K280E^ mutation on SL signaling. **(A)** Subcellular localizations of GFP, D14-GFP and D14^K280E^-GFP in rice protoplasts. The *35S:SV40NLS-mCherry* plasmid was cotransformed to label the nucleus. BF, bright field. Bar = 10 μm. **(B)** *In vivo* interaction between D14-Flag and D3-GFP revealed by co-IP assay in rice protoplasts. After transformation and incubation for 12 h, protoplasts were treated with 10 μM *rac*-GR24 for 1 h, and then the supernatant extracted from the protoplasts was incubated with an agarose-conjugated anti-GFP monoclonal antibody at 4°C for 3 h in the presence or absence of 10 μM *rac*-GR24, following which the D14 recombinant protein was detected with an anti-Flag monoclonal antibody, while D3-GFP and GFP were detected with an anti-GFP monoclonal antibody. Input refers to the total protein lysate before immunoprecipitation. **(C)** D53 protein levels in calli of *Act:D14-GFP/d14*, *Act:D14^K280E^-GFP/d14* and *d14* treated with 10 μM GR24. D53 was detected by immunoblotting with anti-D53 polyclonal antibodies. Rice Actin1 contents were used as loading controls in the immunoblotting analyses.

We then evaluated whether the K280 mutation influence the function of D14 in SL signaling. It has been reported that AtD14 is localized in the nucleus and cytoplasm (Chevalier et al., 2014). We therefore investigated the subcellular localization of D14*-*GFP and D14^K280E^*-*GFP in a transient expression system using the SV40NLS-mCherry as a nuclear marker (Wang et al., 2015). As shown in **Figure 3A**, both D14-GFP and D14^K280E^-GFP proteins were localized in cytoplasm and nucleus, suggesting that K280 mutation does not affect the subcellular localization of D14. We then tested the interaction between D14^K280E^ and D3 in wild-type protoplasts using the co-IP assay and found that D14^K280E^ could interact with D3 after *rac-*GR24 treatment (**Figure 3B**). Furthermore, we compared the SL-induced degradation of D53 in calli of *d14*, *D14-GFP/d14* and *D14^K280E^-GFP/d14*. The protein levels of D14 and D14^K280E^ were comparable in different transgenic lines and displayed no obvious decrease after *rac-*GR24 treatment for 30 min (Supplementary Figure 3). Consistent with our previous study (Jiang et al., 2013), D53 degradation upon *rac-*GR24 treatment is dramatically impaired in *d14* mutant. Importantly, both D14*-*GFP and D14^K280E^*-*GFP rescue the defect of D53 degradation in *d14*, indicating that D14^K280E^*-*GFP could trigger SL-induced D53 degradation and potential SL signaling (**Figure 3C**). Collectively, the D14^K280E^ mutation doses not disturb the process of SL perception, which requires D14-D3 interaction and D53 degradation.

### SL-Induced D14 Degradation Requires the Hydrolase Activity of D14

**FIGURE 4:**
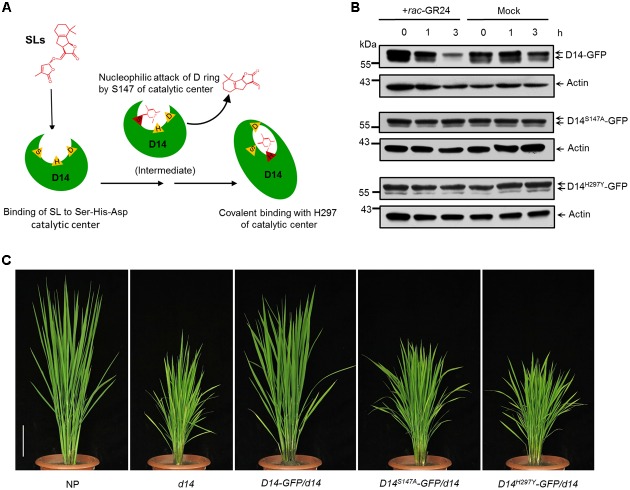
Requirement of hydrolase activity in SL-induced D14 degradation. **(A)** The schematic diagram of SL perception by D14. The open state D14 binds SLs with the Ser-His-Asp catalytic pocket (triangles). The Ser residue (S147) is involved in nucleophilic attack of the D-ring of SLs to initiate the catalytic reaction. Then the His residue (H297) covalently binds the aldehyde carbonyl of the S147-bound moiety to induce the conformational change of D14 from the open state to the close state. **(B)** Protein levels of D14-GFP, D14^S147A^-GFP and D14^H297Y^ -GFP in transgenic calli after the 20 μM *rac-*GR24 or DMSO treatment. The D14-GFP fusion protein was detected with an anti-GFP monoclonal antibody. Actin1 contents were used as loading controls in the immunoblot analyses. **(C)** The complementation analyses of the *d14* mutant with D14-GFP, D14^S147A^-GFP and D14^H297Y^-GFP. Bar = 10 cm.

*D14* encodes a member of the α/β hydrolase superfamily, which features the canonical triad catalytic center (Hamiaux et al., 2012; Kagiyama et al., 2013). The 147th serine and the 297th histidine sites are essential for the hydrolase activity and SL perception of D14 (**Figure 4A**) (Zhao et al., 2013; Yao et al., 2016). To address whether the hydrolase activity of D14 is required for its degradation, we mutated these key amino acids and generated the *D14^S147A^-GFP* and *D14^H297Y^-GFP* overexpression transgenic lines in the *d14* background. Compared to the *Actin:D14-GFP/d14* transgenic calli, the SL-stimulated D14 degradation was severely inhibited in *Actin:D14^S147A^-GFP/d14* and *Actin:D14^H297Y^-GFP/d14* calli (**Figure 4B**), indicating that the hydrolase activity of D14 is essential for the SL-induced D14 degradation. More importantly, the *Actin:D14^S147A^-GFP/d14* and *Actin:D14^H297Y^-GFP/d14* transgenic plants exhibited dwarf and high tillering phenotypes as the *d14* mutant (**Figure 4C**), demonstrating that the S147 and H297 sites are both indispensable for the signal perception of SLs *in vivo*.

### SL-Induced D14 Degradation Is Dependent on D3 and Coupled to D53 Degradation

**FIGURE 5:**
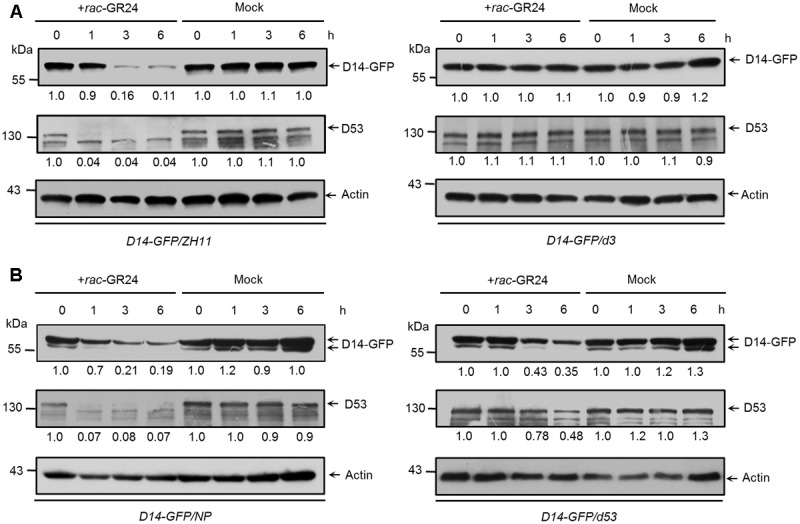
The SL-triggered D14 degradation in *d3* and *d53* mutants. **(A)** D14-GFP and D53 protein levels in *Act:D14-GFP* transgenic calli in the wild-type (ZH11) or *d3* background after 20 μM *rac*-GR24 or DMSO treatment. **(B)** D14-GFP and D53 protein levels in transgenic calli of wild-type (Nipponbare) or *d53* background after 20 μM *rac*-GR24 or DMSO treatment at different time points. D14-GFP and D53 levels were detected by immunoblotting with an anti-GFP monoclonal antibody and anti-D53 polyclonal antibodies, respectively. Relative amounts of proteins were determined by densitometry and normalized to actin1 and expressed relative to the value at zero time. Actin1 contents were used as loading controls in all the immunoblotting analyses.

*D3* encodes an F-box protein, which is a subunit of the Skp-Cullin-F-box (SCF) complex and is responsible for substrate recognition (Smith and Li, 2014). SLs trigger the interaction between D14 and D3, which in turn leads to the ubiquitination and degradation of D53, and probably relieves the repression on gene expression (Jiang et al., 2013; Zhou et al., 2013). To check whether the degradation of D14 is depended on D3, we first identified a loss-of-function mutant of *d3* in the ZH11 background, which contains a premature translation mutation due to a 1-bp (base pair) deletion in the first exon and displayed dwarf and high-tillering phenotypes (Supplementary Figure 4). We then treated the *Actin:D14-GFP* transgenic calli in the wild-type or *d3* background with 20 μM *rac*-GR24. Consistent with the role of D3 in targeting D53 for ubiquitination and degradation, we found that D53 degradation upon SL treatment was strongly inhibited in the *d3* mutant. More importantly, D14 degradation was also blocked in the *d3* mutant (**Figure 5A**), suggesting that D3 may directly involves in the proteolysis of D14 or D3 regulates the expression of unknown SL-responsive genes and consequently control D14 degradation.

Furthermore, we compared the amounts of D14-GFP and D53 in *Actin:D14-GFP* transgenic calli in the wild-type and *d53* background (**Figure 5B**). The *d53* mutant shows typical SL-deficient phenotypes including dwarf and high tillering, and is insensitive to exogenous SL treatment. These developmental defects are caused by a significant attenuation of D53 degradation due to an in-frame deletion of amino acid 813-817 and an amino acid substitution at 812 of D53 protein (Jiang et al., 2013). Thus the SL signaling is impaired in the *d53* mutant. After *rac*-GR24 treatment for 1 h, endogenous D53 protein almost disappeared in wild type (left) but remained stable in the *d53* mutant (right), consistent with results in **Figure 3C** and our previous observation that D53 was almost disappeared within 30 min but remained stable in *d53* (Jiang et al., 2013). Meanwhile, the protein levels of D14-GFP in wild type began to decrease (left) but remained stable in *d53* (right). With extended SL treatment for 3 and 6 h, endogenous D53 in the *d53* mutant began to degrade and reached about half of the value at zero time. The D14-GFP also degraded in *d53* (right) but the degradation degree was significantly impaired compared to that of wild type (left) (**Figure 5B**). These results indicate that D14 degradation is coupled to D53 degradation, which is closely related to the status of SL signaling.

## Discussion

**FIGURE 6:**
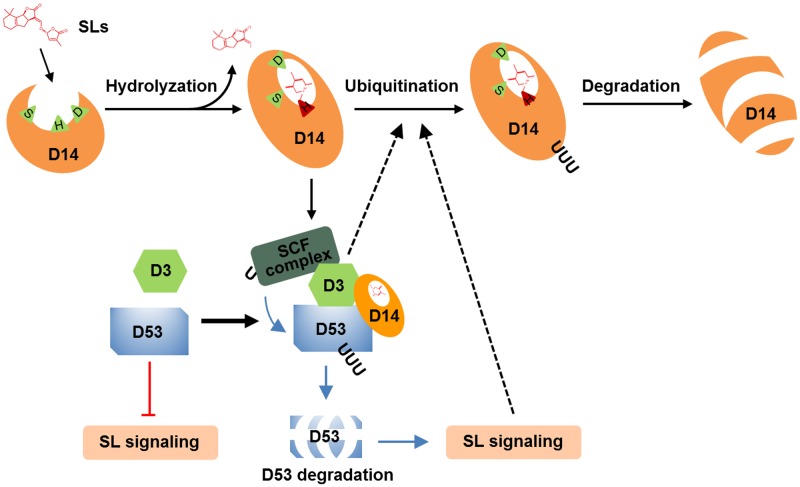
A proposed mechanism of D14 degradation in rice. D14 binds, attacks and hydrolyses SLs into ABC-ring and D-ring-derived molecules, covalently binds the D-ring-derived molecule, and forms a complex with D3 and D53. D53 undergoes ubiquitination and degradation, which relieve the repression on SL signal transduction. Subsequently, D14 is ubiquitinated and degraded through unknown mechanism to down regulate or shut down the SL signal transduction. D3 or the downstream targets of SL signaling may involve in this important feedback regulation.

The degradation of receptors exists as a fine-tune mechanism in the signaling of hormones, such as ABA, JA, brassinolide and ethylene (Kevany et al., 2007; Wu et al., 2011; Yan et al., 2013; Kong et al., 2015). In this study, we found that D14 degradation was observed from 1 h and reached maximum level at about 3–4 h (**Figures 1A,C, 4B, 5**), while D53 protein is degraded at 10 min and almost disappeared at 30 min after GR24 treatment (**Figure 3C**), indicating that the SL receptor D14 is degraded through the 26S proteasome following the degradation of D53 in rice. SLs are recognized and hydrolyzed into a D-ring-derived molecule by D14 and form a covalently link bridge with the catalytic sites of D14 (Yao et al., 2016). This process stimulates a conformational change of D14 and the formation of a SCF^D3^-D14-D53 complex, leading to the ubiquitination and degradation of D53 (Jiang et al., 2013; Zhou et al., 2013). Subsequently, the repression of D53 on SL signaling is relieved, and the downstream components are activated to regulate plant development. Meanwhile, SLs could in turn trigger the degradation of D14 through the 26S proteasome pathway, leading to the inactivation of SL perception (**Figure 6**).

The molecular mechanisms by which D14 was ubiquitinated and degraded are still open questions in higher plants (Chevalier et al., 2014; Wang et al., 2017). We found that D14 degradation was blocked in the loss-of-function mutant of *d3* that displays completed obstructed D53 degradation, and was impaired in the dominant mutant *d53* that displays significantly attenuated D53 degradation (**Figure 5**). These phenomena raised two possibilities for D14 degradation *in vivo*. The F-box protein D3 may target D53 and then D14 for ubiquitination and degradation. In this case, the over-accumulated D53 protein in the *d53* mutant may hold more D3 protein and disturb the ubiquitination of D14. But another scenario also fit the available information. An unknown E3 ligase downstream of SL signaling may involve in the ubiquitination and degradation of D14. When SL perception is blocked (as in *d3* mutant) or attenuated (as in *d53* mutant), the expression or modification of this E3 ligase may change accordingly and thus influence D14 degradation (**Figure 6**). Crucially, further analysis of D14 ubiquitination and degradation based on *in vitro* reconstitution of SCF^D3^ E3 ligase activity and the structural analysis of D14-D3-D53 complex will give direct evidence of whether D3 direct target D14 for ubiquitination and degradation.

Feedback regulation is an important and universal mechanism to maintain the homeostasis of a signaling pathway (Schwechheimer, 2008). In plants, the biosynthesis and signal transduction of hormones are adjusted by several transcriptional and post-translational feedback regulation (Benjamins and Scheres, 2008; Kendrick and Chang, 2008; To and Kieber, 2008; Vlot et al., 2009; Jaillais and Chory, 2010; Jaillais and Vert, 2016; Yu et al., 2016; Carvalhais et al., 2017). For examples, auxins trigger the degradation of repressor proteins AXIN/INDOLE-3-ACETIC ACIDs (Aux/IAAs) and subsequently release the AUXIN RESPONSE FACTOR (ARF) transcription factors to regulate signal transduction. However, ARF could also promote the expression of *Aux/IAA* genes to repress the auxin signaling (Benjamins and Scheres, 2008). In the SL biosynthetic pathway, SLs repress the transcription of *MAX3* and *MAX4*, which are key biosynthetic genes in Arabidopsis (Mashiguchi et al., 2009; Wang et al., 2015). In addition, in *max* and *d* mutants, the expression levels of *MAX4* and its rice orthologous gene *D10* were up-regulated (Jiang et al., 2013; Zhou et al., 2013; Wang et al., 2015). In the SL signaling pathway, SLs trigger rapid degradation of D53 to promote SL signaling in rice, but in turn activate the transcription of the *D53* gene to limit SL signaling (Jiang et al., 2013; Zhou et al., 2013). This feedback regulation is mediated by the transcription factor IPA1, which could interact with D53 and activate *D53* expression through directly binding the *D53* promoter (Song et al., 2017). In Arabidopsis, AtD14 has been reported to undergo SL-induced and MAX2-dependent degradation through the proteasome system (Chevalier et al., 2014). In this work, we showed that SLs also promote the D14 degradation in rice, which would form a negative feedback loop of SL signaling. Intriguingly, SLs could trigger D53 degradation from 5 min, then downregulate *MAX3* and *MAX4* expression levels while upregulate the *D53* expression from about 1–2 h. The SL-induced D14 degradation was observed from 1 h and reached maximum level at about 3–4 h, suggesting that the precise feedback regulation loops in different time dimension would effectively modulate the duration and intensity of SL signaling.

Parasitic plants are the largest biotic cause of reduced crop yields throughout the Africa (Rodenburg et al., 2016; Gobena et al., 2017). The germination of parasitic plants, mainly including *Striga* and *Orobanche*, is triggered by SLs secreted from crop roots (Waters et al., 2017). Currently, several strategies have been proposed to control parasitic plants, including induction of *Striga* germination through treating the unplanted fields with SL-like compounds and kill the parasitic plants before planting crops, and breeding of new crop varieties with altered SL biosynthesis or secretion without harming their normal growth (Nakamura and Asami, 2014; Toh et al., 2015; Fernández-Aparicio et al., 2016; Holbrook-Smith et al., 2016). Recent studies on highly *Striga*-resistant sorghum lines indicated that the SL profiles of root exudates from the *lgs1* variants displayed reduced 5-deoxystrigol, a highly active *Striga* germination stimulant, but enhanced orobanchol, an SL required for normal growth. This change did not bring disadvantageous effect on plant development, but could inhibit the stimulation of *Striga* germination effectively (Gobena et al., 2017). In this study, we found that the mutation of D14^K280E^ did not affect the normal function of D14 and SL signaling, but inhibited the D14 ubiquitination and degradation following SL treatment (**Figures 2, 3**). It is speculated that overexpressing the *D14^K280E^* may lead to the hypersensitivity to SLs but this may also need other amino acids working cooperatively with K280, for example K33, which moderately regulate D14 degradation (**Figure 2C**). Furthermore, when the mutated D14 protein resistant to ubiquitination and degradation is overexpressed, tiller number in rice is speculated to decrease due to an enhanced SL perception. Overexpression of this kind of D14 protein may suppress the high tillering phenotypes *d27* and *d17* that display impaired SL biosynthesis. This may present an alternative approach to cultivate rice varieties that perform decreased SL biosynthesis and reduced germination of *Striga* without harming the internal SL signaling pathway and regular plant development. It is rational that genetic modifications on the biosynthesis and perception of SLs are applicable to control the parasitic plant growth in crops in the future.

## Author Contributions

QH, YH, BW, and JL conceived this project and designed all experiments. QH, YH, LW, SL, XM, GL, YJ, MC, XS, and LJ performed some of the experiments. BW, QH, YH, HY, and JL analyzed the data and wrote the paper. All authors commented on the article.

## Conflict of Interest Statement

The authors declare that the research was conducted in the absence of any commercial or financial relationships that could be construed as a potential conflict of interest.

## Footnotes

1 http://bdmpub.biocuckoo.org/results.php
