# Supplementary material for: DWARF14, A Receptor Covalently Linked with the Active Form of Strigolactones, Undergoes Strigolactone-Dependent Degradation in Rice
Source: Front Plant Sci. 2017 Nov 9;8:1935. doi: 10.3389/fpls.2017.01935 (PMC5684176; doi:10.3389/fpls.2017.01935)
Supplement: Supplementary file 1 [file Image_1.PDF]

Supplementary Material

DWARF14, A Receptor Covalently Linked with the Active Form of Strigolactones, Undergoes Strigolactone-Dependent Degradation in Rice

Qingliang Hu[1,2,†,] Yajun He[1,2,†], Lei Wang[1,3], Simiao Liu[1], Xiangbing Meng[1], Guifu Liu[1], Yanhui Jing[1], Mingjiang Chen[1], Xiaoguang Song[1], Liang Jiang[1,4] ,Hong Yu[1], Bing Wang[1,*] and Jiayang Li[1,2,*]

[1]State Key Laboratory of Plant Genomics and National Center for Plant Gene Research (Beijing), Institute of Genetics and Developmental Biology, Chinese Academy of Sciences, Beijing, China, [2]University of Chinese Academy of Sciences, Beijing, China, [3]Present address: Agricultural Genome Institute, Chinese Academy of Agricultural Sciences, Shenzhen, China, [4]Present address: Max Planck Institute of Molecular Plant Physiology, Potsdam, Germany.

†QH and YH contributed equally to this work.

*To whom correspondence may be addressed. E-mail: jyli@genetics.ac.cn or bingwang@genetics.ac.cn.

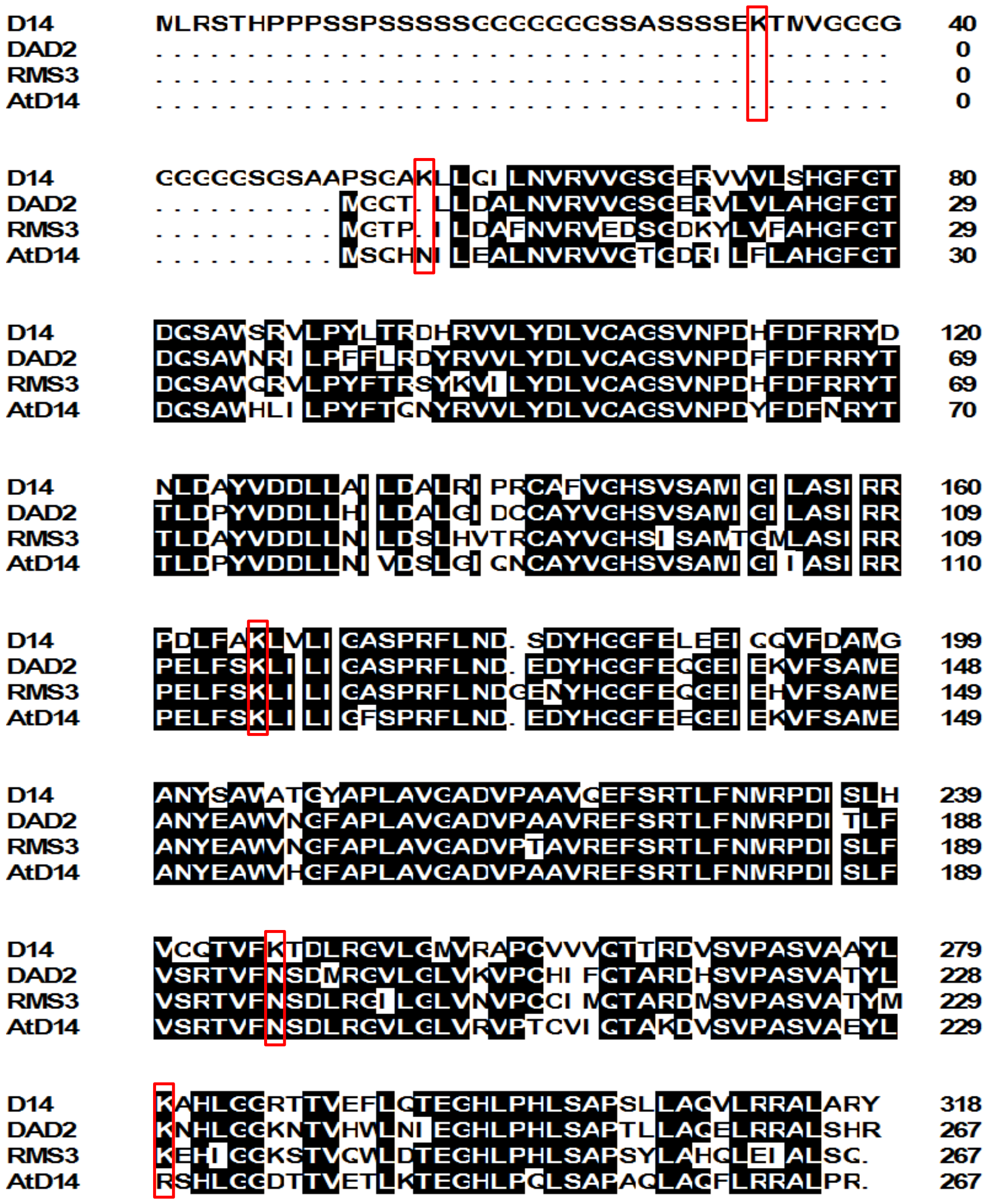

Supplementary Figure 1. Sequence alignment of rice D14, petunia DAD2, pea RMS3, and Arabidopsis D14 (AtD14) by DNAMAN 8.0. Five lysine sites in rice D14 and its homologs are marked by the red box. The GenBank accession numbers for sequences from top to bottom are Q10QA5(D14), AFR68698 (DAD2), AMB61031.1 (RMS3) and Q9SQR3 (AtD14).

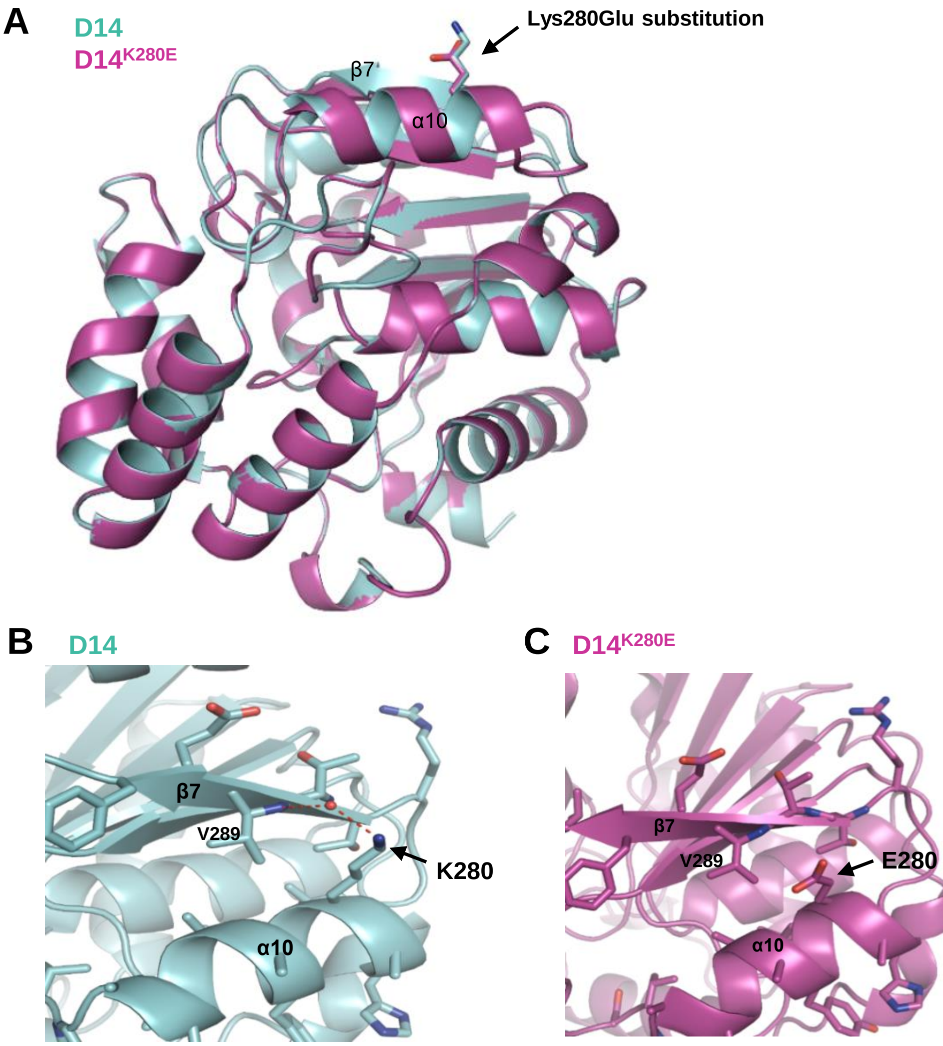

Supplementary Figure 2. The position of K280 in crystal structure of D14. **(A)** Structural superposition of D14 and D14^{K280E}. The structure of D14 was downloaded from PDB (PDB code: 4IH9) and the structure of D14^{K280E} was modelled by SWISS-MODEL website using D14 as template. **(B)** Detailed interaction of K280 in α10 helix with β7 from D14. The residue Lys280 binds to Val289 through the formation of water-mediated (red ball) hydrogen bond. The black arrow showed the residue K280. **(C)** Detailed interaction of E280 in α10 helix with β7 from D14^{K280E}. The residue Glu280 does not form direct hydrogen bonds to Val289. The black arrow showed the residue E280. The green color and pink color represent structures of D14 and D14^{K280E} respectively.

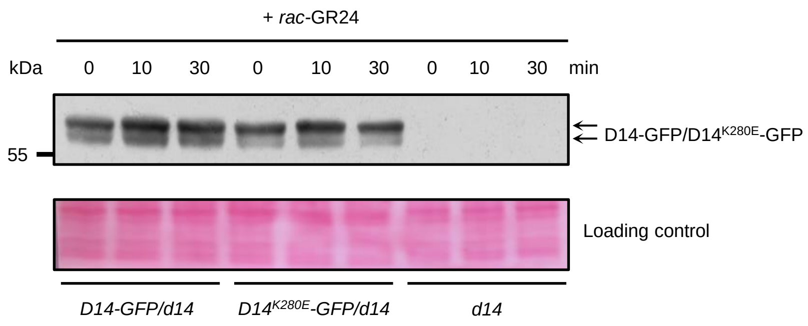

Supplementary Figure 3. The D14-GFP and D14^{K280E}-GFP protein levels in calli of *Act:D14-GFP/d14, Act:D14^{K280E}-GFP/d14* and *d14* treated with 10 µM *rac*-GR24. D14-GFP and D14^{K280E}-GFP were detected by immunoblotting with an anti-GFP monoclonal antibody. Relative protein levels were determined by densitometry and normalized to loadings determined by Ponceau staining (red) in the immunoblotting analyses.

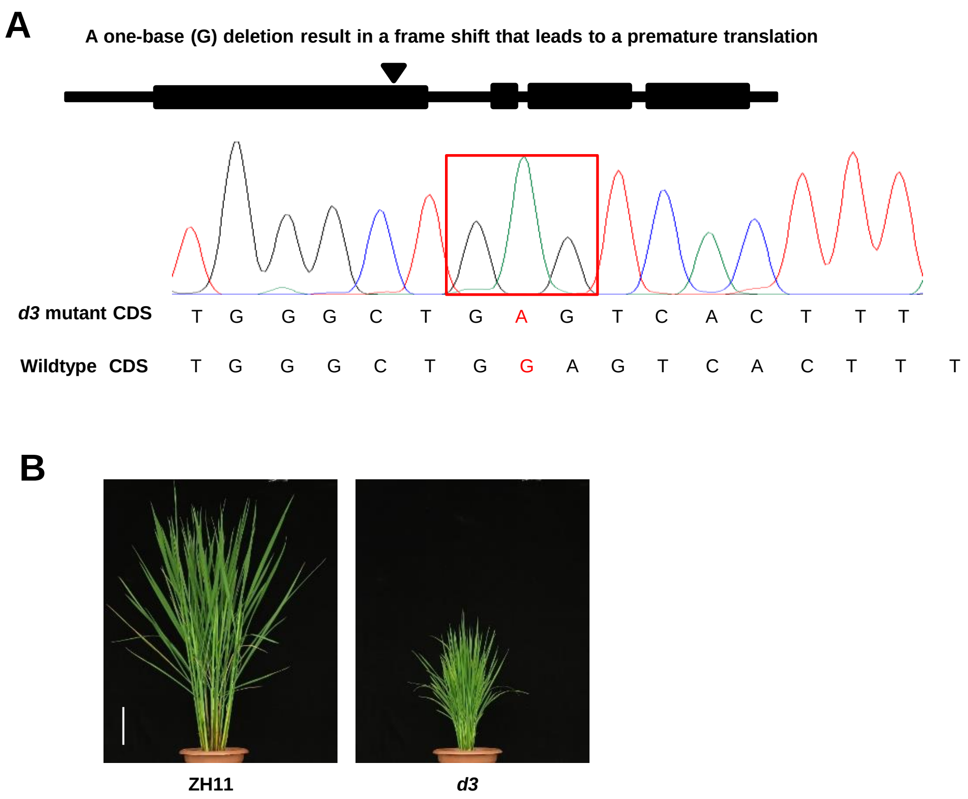

Supplementary Figure 4. Characterization of the *d3* mutant. **(A)** Diagram of *D3* and the mutation site in the *d3* mutant. **(B)** The phenotype of wild type (ZH11) and *d3* at the adult stage (three months). Bar = 10 cm.

Supplemental Table 1. Primers Used in This Study

Primer Name	Sequence (5'-3')
PBI221-D14-F	ACGGGGGACTCTAGAGGATCCATGCTGCGATCGACGCATCC
PBI221-D14-R	GCCCTTGCTCACCATAAGCTTGTACCGGGCGAGAGCGCGGC
35S-D14^{K280E}-F	TCGCCGCCTACCTCGAGGCCCACCTCGG
35S-D14^{K280E}-R	CCGAGGTGGGCCTCGAGGTAGGCGGCGA
Ubi-D14-F	CGACTCTAGAGGATCCATGCTGCGATCGACGCATCC
Ubi-D14-R	AGCTCTCTAGAACTAGTTTACTTGTACAGCTCGTCCA
D14^{K33E}-F	TCGTCGAGCTCGGAGGAGACGATGGTGG
D14^{K33E}-R	CCACCATCGTCTCCTCCGAGCTCGACGA
D14^{K55E}-F	GAGCGGGGCGGAGCTGCTGCAGATCCTG
D14^{K55E}-R	CAGGATCTGCAGCAGCTCCGCCCCGCTC
D14^{K166E}-F	ACCTGACCTCTTCGCCGAGCTTGTCCTC
D14^{K166E}-R	GAGGACAAGCTCGGCGAAGAGGTCAGGT
D14^{K246E}-F	TCTGCCAGACCGTCTTCGAGACCGACCT
D14^{K246E}-R	AGGTCGGTCTCGAAGACGGTCTGGCAGA
D14^{K280E}-F	TCGCCGCCTACCTCGAGGCCCACCTCGG
D14^{K280E}-R	CCGAGGTGGGCCTCGAGGTAGGCGGCGA
qRT-PCR-Ubi-F	AACCAGCTGAGGCCCAAGA
qRT-PCR-Ubi-R	ACGATTGATTTAACCAGTCCATGA
qRT-PCR-D53-F	GAGGAGGATAGGAAACCTGTGCC
qRT-PCR-D53-R	GTCTCCTTTCACTGCTGGTAC
qRT-PCR-D14-F	GTGCTGTCGCATGGCTTC
qRT-PCR-D14-R	GCAGGTCGTCGACGTAGG
